# Measuring biological age to assess colony demographics in honeybees

**DOI:** 10.1371/journal.pone.0209192

**Published:** 2018-12-13

**Authors:** Cedric Alaux, Samuel Soubeyrand, Alberto Prado, Mathilde Peruzzi, Alban Maisonnasse, Julien Vallon, Julie Hernandez, Pascal Jourdan, Yves Le Conte

**Affiliations:** 1 INRA, Abeilles et Environnement, Avignon, France; 2 UMT PrADE, Avignon, France; 3 INRA, BioSP, Avignon, France; 4 ADAPI, Avignon, France; 5 ITSAP-Institut de l’Abeille, Avignon, France; University of California San Diego, UNITED STATES

## Abstract

Honeybee colonies are increasingly exposed to environmental stress factors, which can lead to their decline or failure. However, there are major gaps in stressor risk assessment due to the difficulty of assessing the honeybee colony state and detecting abnormal events. Since stress factors usually induce a demographic disturbance in the colony (e.g. loss of foragers, early transition from nurse to forager state), we suggest that disturbances could be revealed indirectly by measuring the age- and task-related physiological state of bees, which can be referred to as biological age (an indicator of the changes in physiological state that occur throughout an individual lifespan). We therefore estimated the biological age of bees from the relationship between age and biomarkers of task specialization (vitellogenin and the adipokinetic hormone receptor). This relationship was determined from a calibrated sample set of known-age bees and mathematically modelled for biological age prediction. Then, we determined throughout the foraging season the evolution of the biological age of bees from colonies with low (conventional apiary) or high *Varroa destructor* infestation rates (organic apiary). We found that the biological age of bees from the conventional apiary progressively decreased from the spring (17 days) to the fall (6 days). However, in colonies from the organic apiary, the population aged from spring (13 days) to summer (18.5 days) and then rejuvenated in the fall (13 days) after *Varroa* treatment. Biological age was positively correlated with the amount of brood (open and closed cells) in the apiary with low *Varroa* pressure, and negatively correlated with *Varroa* infestation level in the apiary with high *Varroa* pressure. Altogether, these results show that the estimation of biological age is a useful and effective method for assessing colony demographic state and likely detrimental effects of stress factors.

## Introduction

As eusocial insects, honeybees live in societies that are notably characterized by an age-dependent division of labor among workers for tasks related to colony homeostasis, growth and development [1]. Bees generally spend the first 2–3 weeks of their adult life working in the hive (feeding and taking care of the brood, building comb), and then the rest of their life outside of the hive (foraging for nectar and pollen to support colony growth) [2], this task switch being associated with a rapid senescence [3]. However, this behavioral maturation and therefore ageing is flexible and may change according to colony needs [4, 5]. For instance, the transition to foraging is socially regulated, such that the presence of a sufficient foraging force delays the onset of foraging of young bees [6–8]. Colony resilience to environmental changes also relies on a pool of previously inactive individuals that acts as a backup force against environmental fluctuations or bee losses [9]. This pool can be large, and has been estimated to be 50% or more of the colony population [10, 11].

However, in a rapidly changing environment, honeybee colonies are increasingly exposed to diverse sources of stress, which represent a challenge to their social homeostasis and can ultimately lead to their collapse [12, 13]. A multifactorial etiology has often been reported (e.g. new parasites, decline in flower availability/diversity and exposure to agrochemicals, acting in isolation or in combination) [13] but there are still major gaps in the environmental risk assessment of stressors and the implementation of effective policy and management responses. This is notably due to the complex nature of the potential combinations of stressors, but also to the difficulty in assessing the honeybee colony state and abnormal events in a robust manner in ever-changing environmental conditions.

The nurse/forager ratio is crucial to colony functioning and colonies generally respond to perturbations via the plasticity of the division of labor (reversal or acceleration of behavioral maturation). For instance, a significant loss of foragers for the colony will accelerate the behavioral maturation of young bees to replace them [2]. Similarly, most stressors, such as starvation [14, 15] and parasites [16–18] lead to the development of precocious foragers. Bees also have the capacity to exhibit a reversal of the normal task-schedule by switching from forager task to nurse task, especially in the absence of nurse bees in the colony [19]. Therefore, stress exposure may induce a demographic shift in the colony (i.e. change in the nurse/forager ratio), and detecting such demographic changes could provide a new way to assess colony state in field-based studies. This redistribution or imbalance of the nurse/forager ratio could be revealed indirectly by the biological age of individuals [20]. Indeed, biological age, as opposed to chronological age, which is the time elapsed since the birth or emergence of the organism, refers to the changes in the physiological state of an organism that occur throughout its lifespan and is by definition dependent on external factors. For instance, organisms can have the same chronological age but different biological ages depending on their environments (e.g. resource availability, parasite exposure). Biological age, which shows the real biological state of an organism, can be determined by key life-stage events (e.g. age of reproduction, age at onset of foraging in bees) but can also be measured by physiological markers [21].

During their age-dependent shift from nurse to forager life stages, or vice versa, worker bees exhibit changes in many physiological traits, including levels of endocrine activity, metabolism, stress resistance, and stored proteins [22, 23]. We thus propose to use physiological markers that change with age and behavioral specialization to estimate the biological age of bees and variation in colony demography. For that purpose, we first developed a calibration approach to determine the relationship between age and biomarkers, and mathematically modelled this relationship to perform biological age prediction. Then, we used this predictive model to assess variation in the biological age of bees from colonies belonging to apiaries with low or high infestation levels of the parasitic mite *Varroa destructor*, the greatest threat to colonies worldwide [24]. Finally, we looked for variations in colony parameters that could be associated with variation in population biological age. Regarding the physiological markers, we measured the expression levels of vitellogenin (*vg*) and the adipokinetic hormone receptor (*akhr*), which are expressed in the fat body and exhibit opposing expression patterns between nurses and foragers [25, 26]. Vitellogenin, which has a protective role against oxidative stress [27] and is used for jelly production [28], plays a key role in the regulation of behavioral maturation and is more highly expressed in nurses than in foragers [29]. It is also a marker of the state of bee health [30–32]. The adipokinetic hormone receptor in honeybees is involved in carbohydrate mobilization [33] and is more highly expressed in foragers than in nurses [25, 26].

## Materials and methods

### Experiment 1: Biological age markers

#### Bee sampling

The goal of this experiment was to determine the relationship between age and physiological markers related to the age-dependent division of labor. This experiment was performed from mid-May to mid- June 2014 at the Institut National de la Recherche Agronomique (INRA) in Avignon (France) with honeybees (*Apis mellifera*) from the local apiary. To develop calibration sample sets of known-age bees, we sampled age-matched bees from eight colonies by placing honeycombs containing late-stage pupae into an incubator at 34° C and 50–70% humidity, and collected individuals that emerged within 12 hours. Bees were then mixed and given a paint mark on the thorax (Posca pen, Japan) before being introduced into four host colonies. Each host colony received around 1,200 paint-marked bees. Early in the morning, pools of 40 paint-marked bees per colony were collected on brood frames, on which all age cohorts can be normally found [34, 35]. Bees were sampled at the age of 0 (newly-emerged bees), 2, 4, 7, 10, 13, 17, 21 and 24 days and immediately plunged in liquid nitrogen. They were then stored in the lab at -80°C until gene expression analysis (see below).

#### Biological age model

We modelled the relationship between age and biomarkers (expression levels of *vg* and *akhr*, see below). Age-biomarker data were used to build a semiparametric stochastic model where the response variables are the log2 of the expression levels *vg* and *akhr* and the explanatory variable is the age. In this model, the variables *vg* and *akhr* are assumed to follow independent normal distributions with means *μ*_*vg*_*(age)* and·*μ*_*akhr*_*(age)* and standard deviations *σ*_*vg*_*(age)* and *σ*_*akhr*_*(age)*, which are non-parametrically estimated to avoid making strong assumptions about the forms of the mean and standard-deviation functions. The non-parametric estimation of these means and standard deviations is made as follows: Let index *b* stands for biomarker *vg* or *akhr*; If age is one of the observed ages, such as *age*_*obs*_ in {0,2,4,7,10,13,17,21,24}, then *μ*_*b*_*(age)* is equal to the average of the biomarker levels observed at *age*_*obs*_, and *σ*_*b*_*(age)* is equal to a positive constant *c* plus the standard deviation of the biomarker levels observed at *age*_*obs*_. The constant *c* is an over-dispersion parameter that will account for additional sources of variability when dealing with monitored colonies. If *age* is not one of the observed ages, then *μ*_*b*_*(age)* and *σ*_*b*_*(age)* are simply obtained by linear interpolations between the closest lower and higher values of *μ*_*b*_*(age*_*obs*_*)* and *σ*_*b*_*(age*_*obs*_*)*, respectively.

### Experiment 2: Colony monitoring and biological age of bees

#### Colony monitoring

The goals of this experiment were to *i)* determine the evolution of the biological age of a bee population during the foraging period, *i*.*e*. between spring and fall, and *ii)* determine whether population biological age is influenced by *Varroa* infestation level and colony parameters. For that purpose, in 2014, we followed two apiaries composed of 40 colonies each and managed by professional beekeepers in the Vaucluse (southeast France). Honeybee colonies (*Apis mellifera*) were maintained in Langstroth hives. To obtain apiaries with different *Varroa* infestation levels, colonies in the first apiary received conventional treatments (Apistan) against the parasitic mite *Varroa destructor* (conventional apiary) and colonies from the second apiary were treated with thymol (organic apiary). Previous observations showed that colonies managed with thymol treatments generally exhibit higher *Varroa* infestation rates [36] (personal observation also and see the results). In both apiaries, treatments were performed in mid-August and colonies were managed in the same way the previous years.

To assess the biological age of colony population, approximately 200 bees per colony were collected on brood frames at the beginning of April, June, August and October. A brood frame was shaken once above a hive roof to collect bees. Around 100 bees were placed in a 50 ml centrifuge tube and stored in dry ice in the field, and then at -80°C in the lab, for analysing gene expression levels (see below) and determining their biological age. The other half was placed in a plastic zip bag and stored on ice, and then in the lab at -20°C, for determining *Varroa* infestation level. Phoretic *Varroa* mite infestation rates of colonies were assessed for 100 bees, which were washed with soapy water (Teepol) to dislodge mites for counting [37]. Infestation rate was reported as the number of mites per 100 adult bees.

At each sampling date, we also estimated several colony parameters: area of honey and pollen storage, number of open and closed brood cells, and number of adult bees. Each side of each frame was visually inspected and the area covered by each parameter was reported in percentage (one full side = 100%). Since the percentages of foraging bees and bees flying around during colony inspection could not be estimated, the estimation of the number of adult bees mainly captured in-hive bees. Percentages were then converted into dm^2^ of honey and pollen storage, number of open and closed brood cells, and number of adult bees, considering that a full side of a Langstroth frame has a surface of 9.03 dm^2^ and theoretically contains 1,100 bees and 3,100 brood cells [38].

#### Biological age prediction

We then inferred for each colony and sampling date the biological age of bees with approximate Bayesian computation (ABC) [39], using the average expression levels of biomarkers observed from sampled bees as summary statistics, denoted by *VG*_*obs*_ and *AKHR*_*obs*_ (capital letters are used to mark the average). For this analysis, we built a stochastic model taking as input parameters the mean *m* and the shape *s* of the distribution of bee ages, and providing as output the mean expression levels of biomarkers. The mean *m* was *a priori* simulated in a uniform distribution between 0 and 25 days; the shape parameter *s* was *a priori* simulated in a uniform distribution between 0 and 1, such that age distribution is constrained to have an L shape; the age of bee pools was simulated in a gamma distribution with mean *m* and shape parameter *s* (the standard deviation is therefore *m/√s*); expression levels of biomarkers *vg* and *akhr* for each pool of bees were simulated under the normal distributions specified with Experiment 1 (using *c* = 1 to account for over-dispersion), and their averages, *VG*_*sim*_ and *AKHR*_*sim*_, were used as summary statistics and compared with observed average expression levels *VG*_*obs*_ and *AKHR*_*obs*_. For each colony and each observation date, we applied a rejection-ABC algorithm by simulating 10^5^ sets of parameters *(m*,*s)* leading to 10^5^ sets of summary statistics *(VG*_*sim*_, *AKHR*_*sim*_*)* and by keeping the 100 sets of parameters with the lowest squared distance *(VG*_*sim*_*—VG*_*obs*_ *)*^*2*^ *+ (AKHR*_*sim*_*—AKHR*_*obs*_*)*^*2*^. The average of *m* and *s* over these 100 sets of parameters led to the posterior means of these parameters.

This procedure, which was applied to each colony of each apiary and to each observation date, allowed us to present the distribution of the posterior mean of *m* at the colony level.

### Gene expression level

The expression levels of *vg* and *akhr* were determined in 3 pools of 8 abdomens per colony by age group (Experiment 1) and a pool of 30 bees per colony (Experiment 2). In Experiment 1, pools of 8 abdomens were homogenized in 1 ml of Qiazol reagent (Qiagen, Courtaboeuf, France) with a TissueLyser (Qiagen) (4x30 s at 30 Hz). The homogenate was incubated for 5 min at room temperature and after centrifugation (12,000 g for 30 s. at 4°C) 500 μl of the supernatant was used for RNA extraction. In Experiment 2, 3 pools of 10 abdomens were homogenized in 1 ml of Qiazol and after 5 min of incubation and centrifugation as above, the 3 supernatants were pooled (167 μl each, giving a 501 μl supernatant).

One hundred μl of Chloroform (Sigma, St. Quentin Fallavier, France) were added and the solution was incubated for 3 min and centrifuged (12,000 g for 15 min at 4°C). RNA extraction was carried out as indicated in the Qiagen RNeasy kit for total RNA with on-column DNase I treatment (Qiagen). The High capacity RNA to cDNA Kit (Applied Biosystems, Illkirch, France) was used to reverse-transcribe 1,000 ng of RNA per sample. Complementary DNA samples were then diluted ten-fold in molecular grade water.

The expression levels of *vg* and *akhr* were determined by quantitative PCR using a StepOne-Plus Real-Time PCR System (Applied Biosystems) and the SYBR green detection method including the ROX passive reference dye. Three μl of cDNA were mixed with 5μl SYBR Green PCR Master Mix (Applied Biosystems) and 1 μl of forward and reverse primers (10 μmol) of selected genes. The cycle threshold (Ct) values of *vg* and *akhr* were normalized to the geometric mean of the housekeeping genes *actin* and *rps5* using the comparative quantification method (delta Ct method). We used the following sequences of primers: *vg* forward: TTGACCAAGACAAGCGGAACT, reverse: AAGGTTCGAATTAACGATGAA [40]; *akhr* forward: TTGGGCGATCACTGTTTCCT, reverse: GATGATAAGTACAGGCCAAACATTCTAA [25]; *actin* forward: TGCCAACACTGTCCTTTCTG, reverse: AGAATTGACCCACCAATCCA; *rps5* forward: AATTATTTGGTCGCTGGAATTG, reverse: TAACGTCCAGCAGAATGTGGTA [41].

### Statistical analysis

All statistics were run using the R statistical software. The codes for the age-biomarker model and the ABC procedure are available in S1 File with raw data provided in S2 File. Variation in physiological parameters according to age and sampling date was analyzed using a Kruskal-Wallis test followed by multiple pairwise comparisons with Dunn tests and Bonferroni correction. Variations in colony parameters (honey, pollen, capped brood, open brood, adult population, and *Varroa* infestation) in relation to the sampling dates were analyzed via generalized linear mixed effect models (GLMM) by maximum likelihood using the *lme4* package. The colony was considered as a random variable. Generalized linear hypothesis tests as implemented by the *multcomp* package allowed for multiple comparisons between the different time points. The link between biological age and colony parameters was determined using a linear regression analysis.

## Results

### Experiment 1: Biological age markers

**Fig 1:**
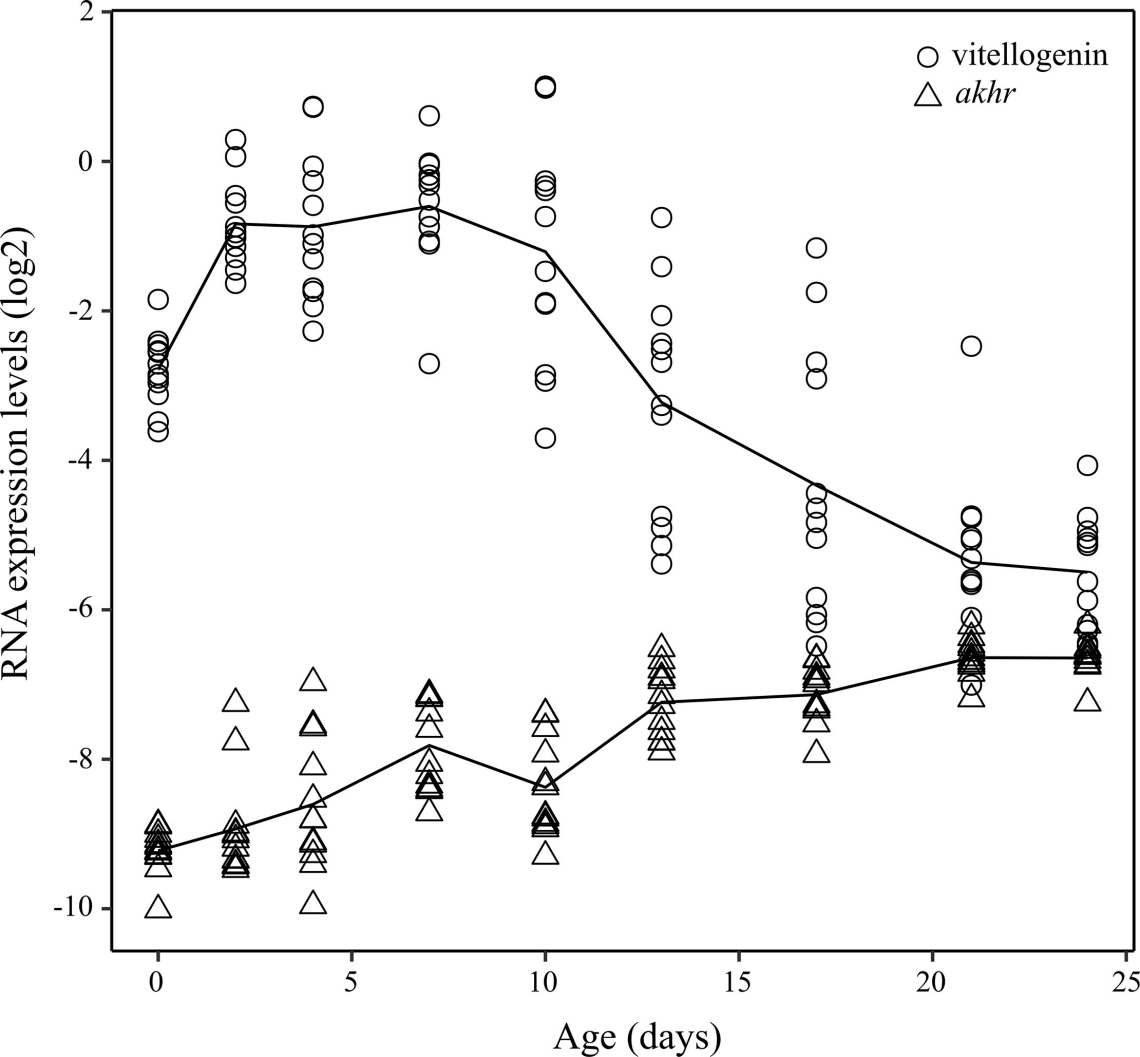
Expression levels of vitellogenin (open circles) and the receptor to adipokinetic hormone (open triangles) as a function of bee age. The solid lines denote average expression levels (n = 12 pools of 8 bees per sampling age).

The level of *vg* significantly changed with age (*H* = 77.28; *df* = 8, 108; *p* < 0.001). It increased sharply after bee emergence, stabilized until day 10 and then decreased until day 20, from which time it remained stable (Fig 1). Inversely, the level of *akhr* increased gradually with age (*H* = 82.81; *df* = 8, 108; *p* < 0.001; Fig 1).

The relationship between age and biomarkers was modelled (S1 File) and an over-dispersion parameter was included to account for additional sources of variability when dealing with colony monitoring (Experiment 2) (S1 Fig). The 95%-confidence envelopes of the model show a good model fit.

### Experiment 2: Colony monitoring and biological age of bees

**Fig 2:**
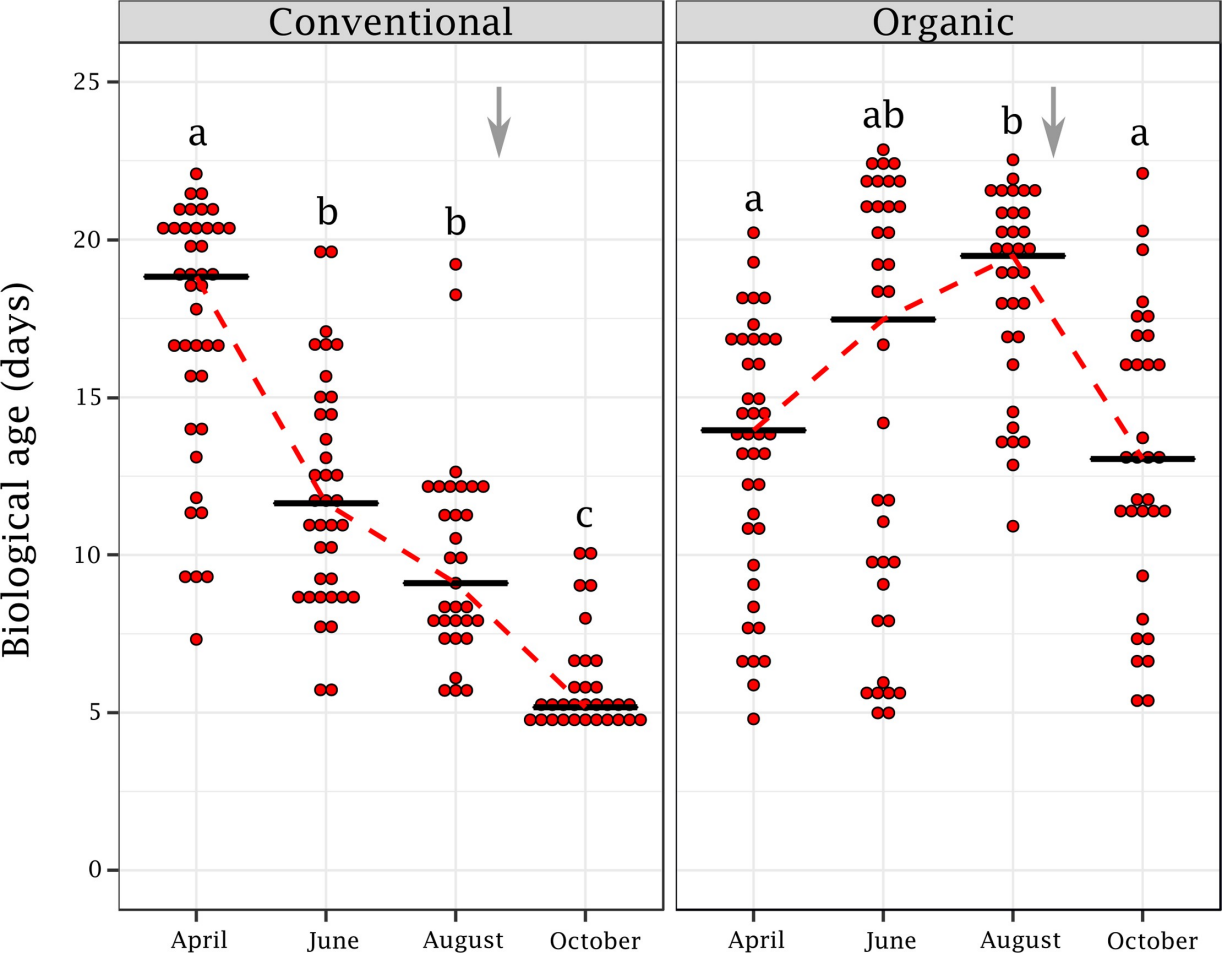
Biological age of colony population from spring to fall. The biological age was predicted from the age model and is shown for the conventional (A) and organic apiaries (B) (n = 31–40 colonies per sampling date and apiary). Different letters indicate significant differences between months (Kruskall–Wallis followed by pairwise Dunn tests with Bonferroni correction). The black line indicates the median biological age. The timing of *Varroa* treatments is indicated by a grey arrow.

Based on *vg* and *akhr* expression levels and the predictive age model (S1 File), we determined the biological age of a colony population. In the conventional apiary, the average age of the population was estimated to be 17 days at the beginning of the spring and progressively dropped to around 6 days before wintering (*H* = 87.18; *df* = 3, 139; *p* < 0.001; Fig 2A). However, in the organic apiary the population aged between April and August (from an average age of 13 to 18.5 days) and then rejuvenated from August to October (around 13 days) (*H* = 25.52; *df* = 3, 141; *p* < 0.001; Fig 2B).

**Fig 3:**
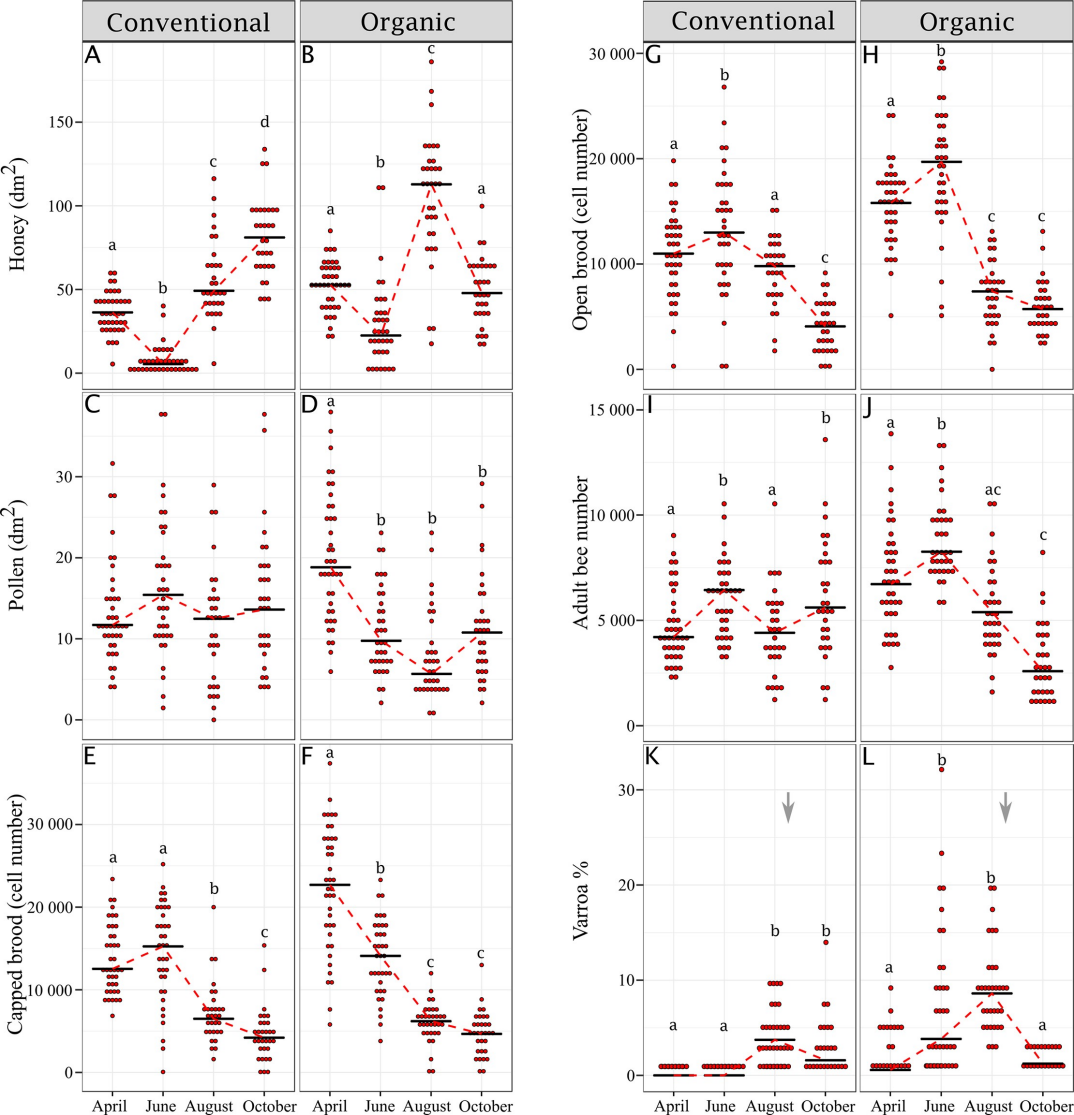
Evolution of colony parameters from spring to fall. Colony parameters (honey (A-B) and pollen storage area (C-D), number of capped (E-F) and open brood cells (G-H), number of adult bees (I-J), and *Varroa* infestation level (K-L)) are shown for the conventional and organic apiaries (n = 31–40 colonies per sampling date and apiary). Different letters indicate significant differences between months (GLMM followed by generalized linear hypothesis tests with Bonferroni correction). The black line indicates the median biological age. The timing of *Varroa* treatments is indicated by a grey arrow.

We then analyzed variations in colony parameters that could be associated with variation in population biological age. Apart from pollen storage in the conventional apiary, each colony parameter changed significantly over the season (Fig 3A–3L). In both apiaries, the level of brood (open and closed cells) was high at the beginning of the foraging season and then declined. While the level of pollen storage remained stable in the conventional apiary, it decreased significantly after April in the organic apiary. *Varroa* infestation level increased between June and August in the conventional apiary but remained rather low (mainly below 5%). In the organic apiary, the infestation level exhibited an earlier and more severe increase until treatments were performed (mid-August for both apiaries).

**Fig 4:**
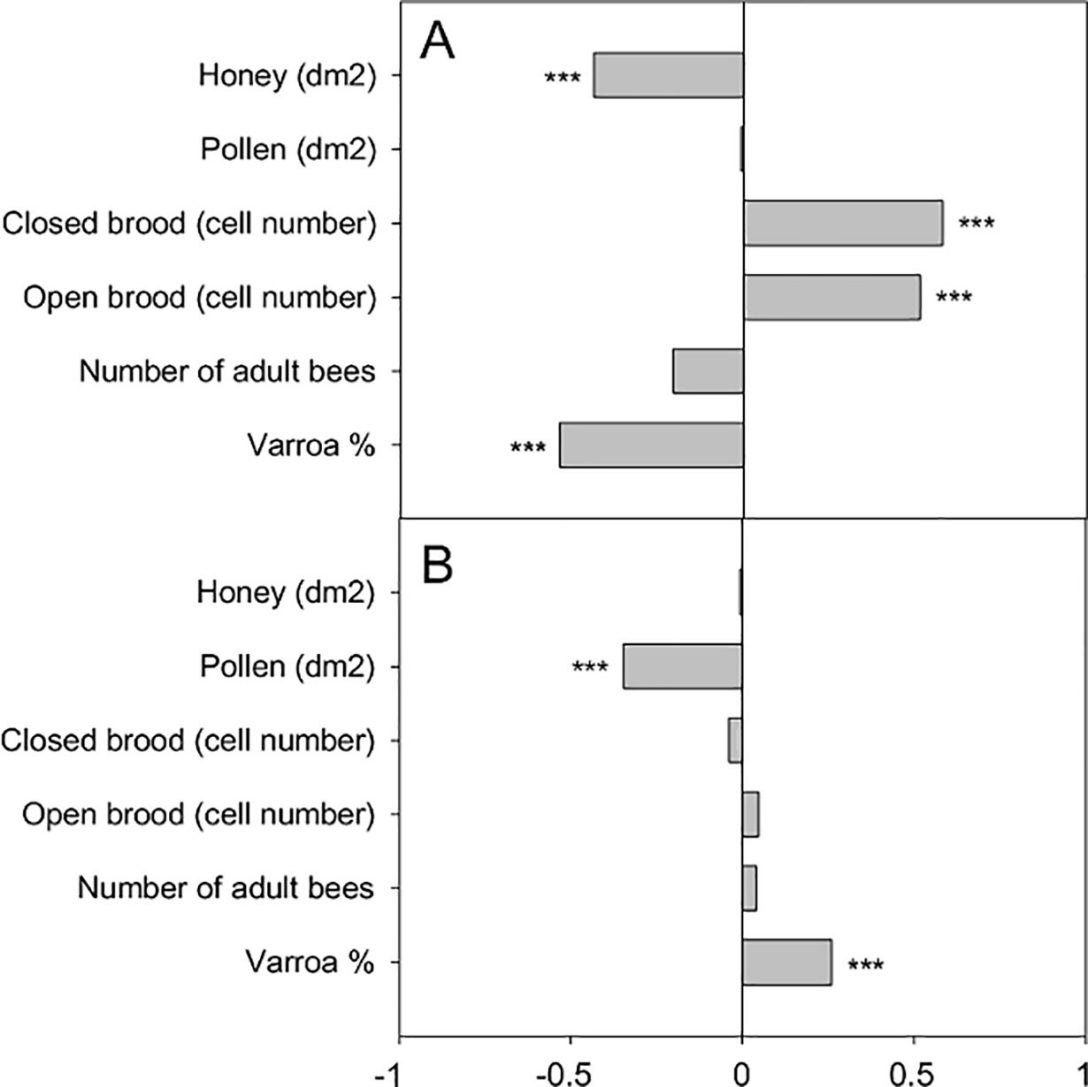
Correlation between biological age and colony parameters. Spearman’s correlation coefficients are shown for the conventional (A) and organic apiaries (B). *** denotes significant correlation (p<0.001).

In the conventional apiary, between April and October, the predicted biological age of the colony population was positively correlated with the amount of brood (open brood cells: *r*^*2*^ = 0.514 and closed brood cells: *r*^*2*^ = 0.579, *p* < 0.001 for both; Fig 4A) but negatively with the amount of stored honey and *Varroa* infestation (*r*^*2*^ = -0.437, *p* <0 .001 and *r*^*2*^ = -0.536, *p* < 0.001, respectively). However, in the organic apiary, the biological age of the colony population was positively correlated with the *Varroa* infestation level (*r*^*2*^ = 0.26, p = 0.002; Fig 4B) and negatively correlated with the amount of stored pollen (*r*^*2*^ = -0.347, *p* < 0.001).

## Discussion

Environmental stress factors can affect colony growth and contribute to colony decline by inducing demographic disturbances. Therefore, we developed a method for assessing colony demographic state, by measuring the biological age of a colony population. We showed that this method is effective for determining changes in colony demography throughout the beekeeping season.

During the foraging period of honeybees (typically between spring and winter), nurse bees might accelerate their behavioral development and forage precociously in response to a loss of foragers or exposure to disease [12, 16–18], and foragers may revert to nurse-activity according to the colony needs (e.g. lack of nurses) [8]. In this context, assessing within-colony demographic changes requires the determination of bee biological age. We found that the markers *vg* and *akhr*, that typically exhibit significant changes during behavioral development [25, 26], are particularly suited for assessing bee biological age and, therefore, colony demographic changes. Markers that change with bee age but do not show consistent differences in expression between nurses and foragers may be better adapted to determining bee chronological age [20]. Since chronological and biological age are uncoupled in honey bees [42], identifying the chronological age of bees may not provide an accurate marker of colony state, at least during the foraging periods, when colony demographics often fluctuate according to changing environment. Markers of chronological age may be better suited for assessing the state of winter bees, which arise during the autumn in temperate regions as an adaptation for surviving through the winter period. During this period there is no population renewal and winter bees, which do not perform brood care or foraging tasks but thermoregulate the colony, can live up to 6 months [43].

In the conventional apiary, a regular decrease in the biological age of colony population was observed between the spring and fall. This observation is consistent with a normal colony life cycle and the changes occurring between foraging periods. At the end of the foraging period (fall), short-lived summer bees are replaced by long-lived winter bees until the next foraging period (spring). The physiological features of winter bees resemble those of nurse bees, with high levels of nutrient storage and vitellogenin [44, 45], until they start to forage at the end of the winter or the beginning of the spring. Furthermore, we found that, within the conventional apiary, variation in the population biological age was positively correlated to the amount of brood (number of open and closed brood cells). Previous studies showed by experimentally manipulating colonies that the removal of brood induced an increase in vitellogenin titer [46, 47]. We therefore can assume that the amount of brood directly affects the biological age of bees: as more brood is produced, the colony population becomes older. Indeed, increased brood rearing requires a greater foraging effort (*i*.*e*. a higher proportion of foragers at the colony level) to sustain the needed resources for consumption by the colony. Conversely, *Varroa* infestation level and the amount of stored honey were negatively correlated with population biological age. This seems counter-intuitive given that *Varroa* infestation is known for decreasing the level of vitellogenin of individual bees [48, 49], and at the colony level *Varroa* infestation rate in the fall is associated with a decrease in *vg* level [30, 32]. However, the *Varroa* infestation levels remained low and mostly below 5% throughout the foraging periods and therefore it is possible that the influence of *Varroa* remained negligible when compared to the influence of the brood. The negative correlation would simply be due to the concomitant growth of the *Varroa* population and a decline in population biological age between spring and fall. A similar explanation could be advanced regarding the link between the amount of stored honey and the biological age of colonies.

Interestingly, we did not observe a decrease in population biological age in the organic apiary. On the contrary, the average biological age increased between April and August, and rebounded to its initial level in the fall. This abnormal population dynamic could be largely explained by the direct influence of *Varroa* on the vitellogenin titer of bees (see above). Indeed, in the organic apiary the *Varroa* infestation level strongly increased until treatments (mid-August). Our data indicates also that a high level of *Varroa* could overcome the influence of other colony parameters like the amount of brood, and therefore prevent the colony from adapting its population demography to its needs. However, since only one apiary per experimental condition was used (conventional and organic), we cannot exclude that variations in colony demography were due to environmental factors (rather than to *Varroa* infestation level). Finally, a negative correlation between the amount of stored pollen and colony biological age was also identified. This could reflect the positive influence of pollen nutrition on *vg* level and therefore the development of a nurse physiological profile [49, 50].

## Conclusions

Our data demonstrated that the biological age of bees can objectively be assessed by measuring biomarkers that are related to the age-dependent division of labor, and thus could be useful for the longitudinal monitoring of colony demography and health. We therefore believe that the ability to gather biological age information in monitoring programs will help to better understand how honeybees respond or adapt to growing environmental pressures. At the same time, further studies, which include larger calibration sample sets (e.g. under different environmental conditions) and combinations of more biomarkers, will improve the predictive power and accuracy of biological markers.

## Supporting information

S1 File Scripts for the age-biomarker model and the ABC procedure. (R)

S2 File Raw data from Experiment 1 (Biological age markers) and Experiment 2 (Colony monitoring and biological age of bees). (XLSX)

S1 Fig Expression levels of vitellogenin (A) and the receptor to adipokinetic hormone (B) as a function of bee age. The solid lines denote the average expression levels (n = 12 pools per sampling age); the dotted lines give the 95%-confidence envelopes of the expression levels with the over-dispersion parameter c equal to 0; the dashed lines give the 95%-confidence envelopes of the expression levels with the over-dispersion parameter c equal to 1 (this value was used in the ABC procedure).
(TIFF)
