# Supplementary figures and images for: Measuring biological age to assess colony demographics in honeybees
Source: PLoS One. 2018 Dec 13;13(12):e0209192. doi: 10.1371/journal.pone.0209192 (PMC6292630; doi:10.1371/journal.pone.0209192)

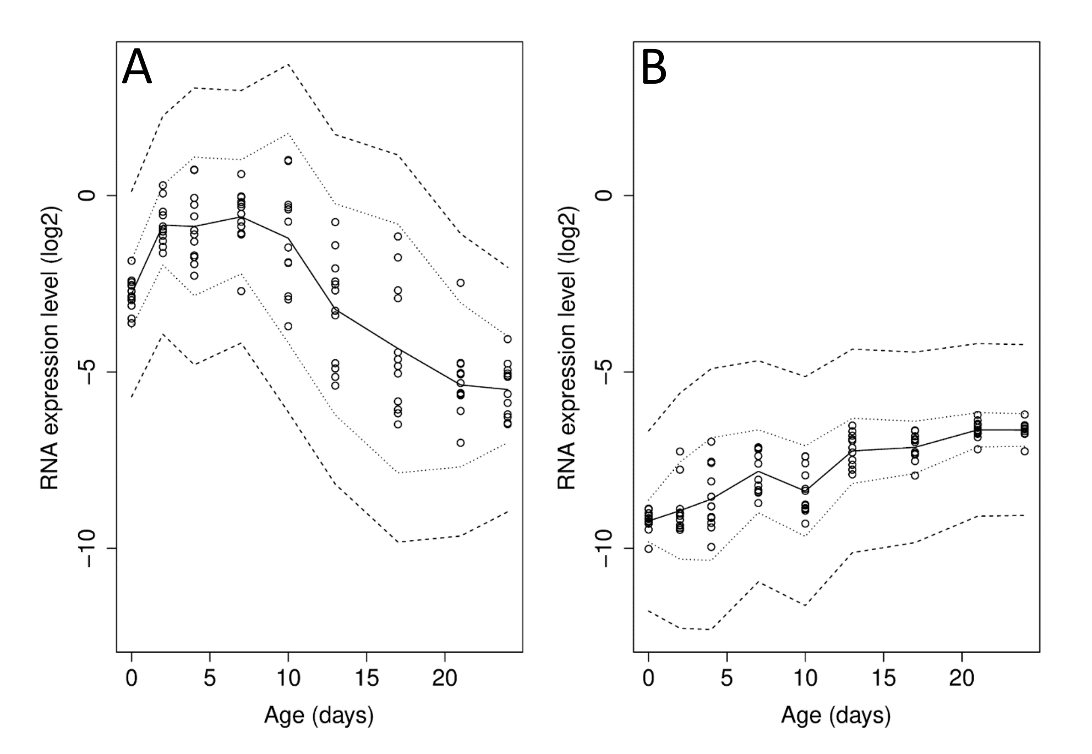

Supplement: S1 Fig — Expression levels of vitellogenin (A) and the receptor to adipokinetic hormone (B) as a function of bee age. The solid lines denote the average expression levels (n = 12 pools per sampling age); the dotted lines give the 95%-confidence envelopes of the expression levels with the over-dispersion parameter c equal to 0; the dashed lines give the 95%-confidence envelopes of the expression levels with the over-dispersion parameter c equal to 1 (this value was used in the ABC procedure). (TIFF) [file pone.0209192.s003.tiff]
